# Dataset on the identification of differentially expressed genes by annealing control primer-based PCR in mitochondrial DNA-depleted myocytes

**DOI:** 10.1016/j.dib.2017.02.016

**Published:** 2017-02-13

**Authors:** Won-Mo Yang, Kyung-Ho Min, Wan Lee

**Affiliations:** a Department of Biochemistry, Dongguk University College of Medicine, Gyeongju 780-714, Republic of Korea; b Endocrine Channelopathy, Channelopathy Research Center, Dongguk University College of Medicine, Goyang 410-773, Republic of Korea

**Keywords:** Mitochondria, Mitochondrial DNA, Differentially expressed genes (DEGs), Annealing controlled primers (ACPs), Myocytes

## Abstract

Changes in the mitochondrial DNA (mtDNA) content are believed to initiate a stress signal that leads to alterations in nuclear gene expression. This article presents data on the identification of nuclear genes that are expressed differentially in response to changes in the mtDNA content in myocytes using annealing controlled primers (ACP)-based PCR technology. The data obtained from L6 GLUT4myc myocytes showed that a total of 19 ACPs produced differentially expressed PCR amplicons in the mtDNA-depleted myocytes. Among those, 13 amplicons were cloned, sequenced, and identified successfully based on the GenBank database. To validate the efficacy of ACP-based PCR analysis, three differentially expressed genes (DEG10, 22 and 26) were confirmed by PCR using the specific primers. The further analysis and detailed results of DEG22 and its functional significance can be found in "C1q tumor necrosis factor alpha-related protein isoform 5 is increased in mitochondrial DNA-depleted myocytes and activates AMP-activated protein kinase." [1].

**Specifications Table**

**Table:** 

Subject area	*Cell Biology, Biochemistry*
More specific subject area	*Gene expression, Metabolism, Mitochondria*
Type of data	*Figure, Table and text*
How data was acquired	*Annealing controlled primer (ACP)-based PCR technology, cloning, sequencing, and RT-PCT*
Data format	*Analyzed*
Experimental factors	*L6 GLUT4myc myocytes were treated with or without EtBr (0.2 μg/ml) and uridine (50 μg/ml) for 3 weeks.*
Experimental features	*Differentially expressed genes (DEGs) in the mtDNA-depleted myocytes were identified using ACP-based PCR, cloning, and sequencing.*
Data source location	*Dongguk University School of Medicine, Gyeongju 780-714, Korea*
Data accessibility	*The data are supplied with this article*

**Value of the data**

• DEGs were analyzed in the L6 GLUT4myc myocytes depleted of mtDNA using an ACP-based PCR technology.
• This data allows a prediction of the biological significance of the cellular mtDNA content in various diseases associated with dysregulation of the mitochondria and metabolism.
• The data reported in this article are useful for comparisons with microarray analysis from other cell or tissue types with depletion of the mtDNA and mitochondrial dysfunction.
• DEGs in this data set could be applied to further studies of the cellular phenotype changes by mtDNA-depletion or mitochondrial dysfunction in myocytes.

## 1 Data

**Fig. 1:**
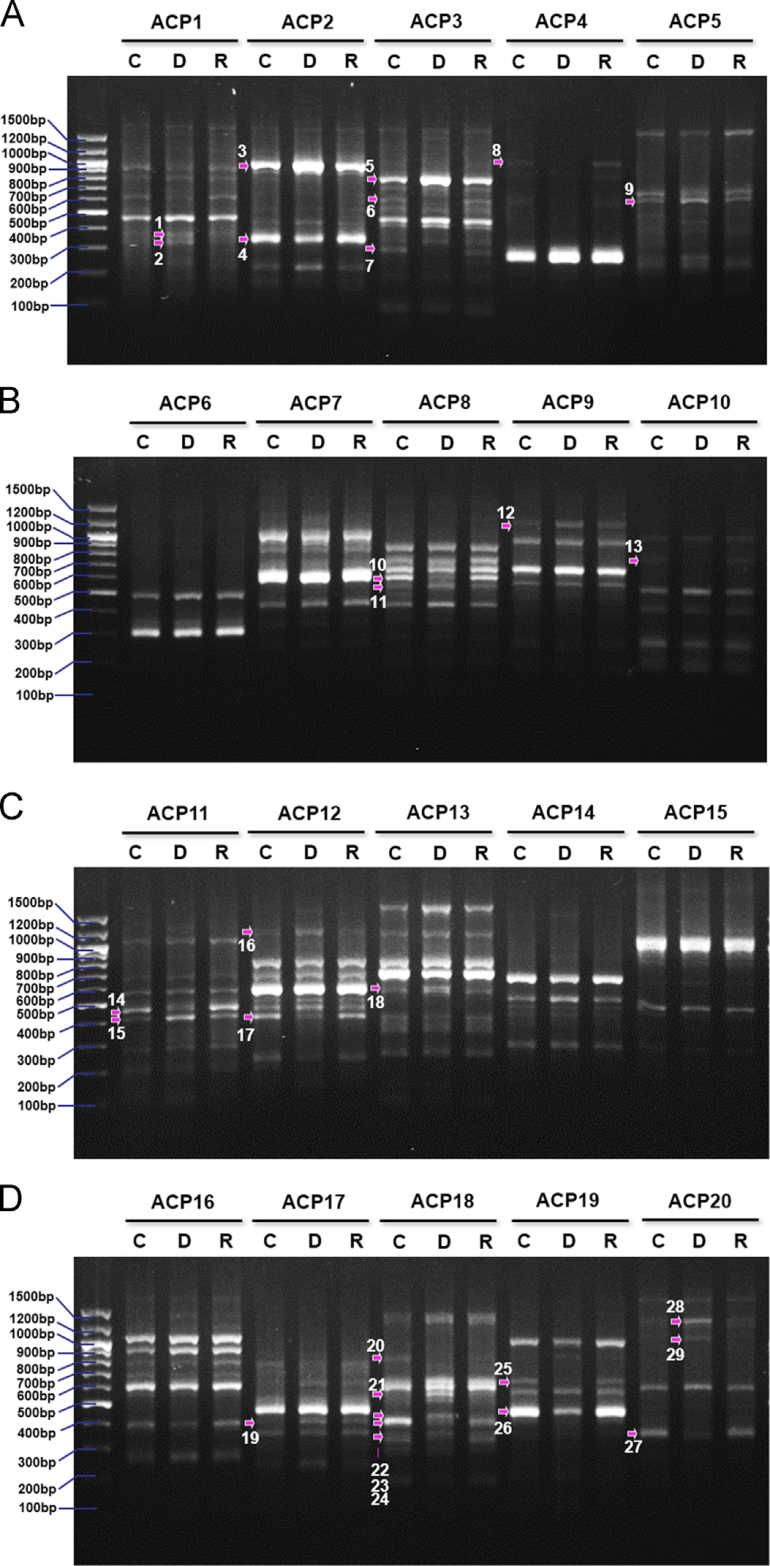
DEGs analyzed by ACP-based PCR in myocytes. Total RNA was extracted from control (C), mtDNA-depleted (D), and -reverted (R) L6 GLUT4myc myocytes and subjected to PCR with various ACPs. The DEGs (arrows numbered) were cloned and sequenced for the further analysis.

**Fig. 2:**
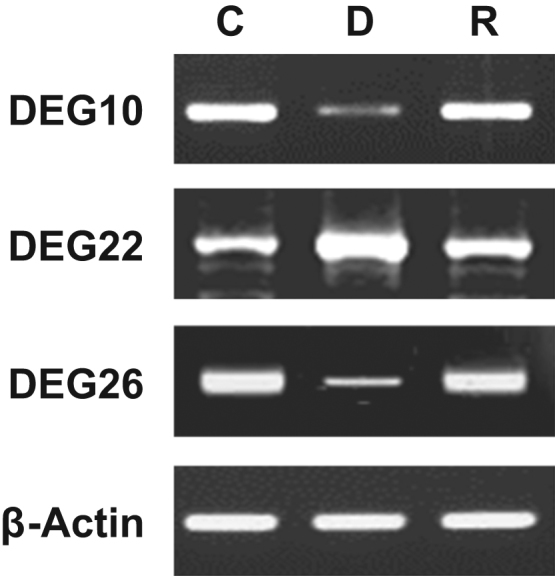
Confirmation of DEGs by RT-PCR. Expression of DEG10, 22 and 26 were confirmed by RT-PCR using the specific primers in control (C), mtDNA-depleted (D), and -reverted (R) L6 GLUT4myc myocytes. β-Actin was used as an internal control.

To identify the differentially expressed genes (DEGs) in the control, mtDNA-depleted, and -reverted L6 GLUT4myc myocytes, annealing control primers (ACP)-based GeneFishing PCR technology was used with a combination of arbitrary ACPs and anchored oligo(dT) primer. Although ACP-based PCR is entirely dependent on primer sets and may not be suitable for genome wide expression profiling, it generates reproducible, accurate, and long (100 bp to 2 kb) PCR products from the identified DEGs. The levels of a number of amplicons were either increased or decreased in the mtDNA-depleted myocytes, as shown in Fig. 1A–D. A total of 19 ACPs exhibited DEGs in the mtDNA-depleted myocytes, with 15 increased DEGs and 14 decreased DEGs compared to the control myocytes. These DEGs were purified from agarose gels and cloned into the TOPO TA cloning vectors for sequence analysis. Among these 29 DEGs, a total of 13 DEGs were sequenced successfully. Table 1 summarizes the identities of 13 DEGs based on the GenBank database analysis. To confirm the efficacy of ACP-based PCR analysis, three DEGs were confirmed by PCR using the specific primers. DEG10, 22, and 26 were verified by RT-PCR, as shown in Fig. 2. Further detailed analysis of DEG22 and its functional significance can be found elsewhere [1].

## 2 Experimental design, materials and methods

### 2.1 Cell culture and mtDNA depletion

The parent cell line used in this study was L6 GLUT4myc, an L6 cell line (provided by Dr. Amira Klip, the Hospital for Sick Children, Toronto, Ontario, Canada) expressing GLUT4-myc, which was constructed by inserting a human c-myc epitope (14 amino acids) into the first ectodomain of rat GLUT4 [2], was used as the parent cell line. The cells were maintained in minimal essential medium-α (MEM-α) supplemented with 10% FBS in a humidified atmosphere containing air and 5% CO_2_ at 37 °C [3]. Changes in the cellular mtDNA content are known to generate retrograde signals from the mitochondria to a range of transcription factors, leading to alterations in nuclear gene expression [4]. As long-term treatments with low concentrations of EtBr are known to inhibit mtDNA replication and transcription selectively without causing changes in the nuclear DNA [5], [6], [7], the L6 GLUT4myc cell line with partially depleted mtDNA was isolated by treating L6 GLUT4myc myocytes with EtBr (0.2 μg/ml) and uridine (50 μg/ml) for 3 weeks in MEM-α supplemented with 10% FBS, and the mtDNA contents in the myocytes were then monitored routinely by amplifying the genomic DNA [8]. The control parental L6 GLUT4myc myocytes were maintained for the same time period under normal culture conditions.

### 2.2 RNA preparation

The total RNA was extracted using the standard Trizol RNA isolation protocol (Life Technologies, Inc., Grand Island, NY, USA). Briefly, 1 ml of Trizol reagent was mixed with a cell pellet (5×10^5^ cells) by repeated pipetting. DNA and protein were excluded by chloroform phase separation. RNA in the aqueous phase was precipitated with isopropanol and resuspended in DEPC-treated water. The quality and quantity of the isolated RNA were determined by the absorbance at 260 and 280 nm.

### 2.3 First-strand cDNA synthesis

The total RNAs extracted from myocytes were subjected to synthesis of the first-strand cDNAs by reverse transcription. Reverse transcription was performed for 1.5 h at 42 °C in a final reaction volume of 20 μl containing 3 μg of the purified total RNA, 4 μl of 5 x reaction buffer (Promega, Madison, WI, USA), 5 μl of dNTPs (each 2 mM), 2 μl of 10 μM dT-ACP1 (5′-CGTGAATGCTGCGACTACGATIIIIIT(18)-3′), 0.5 μl of RNasin, RNase Inhibitor (40 U/μl; Promega), and 1 μl of Moloney murine leukemia virus reverse transcriptase (200 U/μl; Promega). The first-strand cDNAs were diluted by the addition of 80 μl of ultra-purified water for GeneFishing PCR, and stored at −20 °C until needed.

### 2.4 ACP-based PCR

The differentially expressed genes were analyzed by ACP-based PCR using GeneFishing kits (Seegene, Seoul, Republic of Korea) according to the manufacturer׳s instructions. Briefly, the second-strand cDNA was synthesized at 50 °C during one cycle of first-stage PCR in a final reaction volume of 20 μl containing 50 ng of diluted first-strand cDNA, 1 μl of dT-ACP2 (10 μM), 1 μl of 10 μM arbitrary ACP, and 10 μl of 2×Master Mix (Seegene). The sequences of arbitrary ACPs are shown in Table 3. The PCR protocol for second-strand synthesis was a single cycle at 94 °C for 1 min, followed by 50 °C for 3 min, and 72 °C for 1 min. After the synthesis of second-strand DNA, the second-stage PCR amplification protocol was 40 cycles of 94 °C for 40 s, followed by 65 °C for 40 s, 72 °C for 40 s, and a 5 min final extension at 72 °C. The amplified PCR products were separated in 2% agarose gel stained with EtBr.

### 2.5 Cloning and sequencing

The differentially expressed bands were extracted from the gel using the GENCLEAN II Kit (Q-BIO gene, Carlsbad, CA, USA), and cloned directly into a TOPO TA cloning vector (Invitrogen, Carlsbad, CA, USA) according to the manufacturer׳s instructions. The cloned plasmids were sequenced using an ABI PRISM 3100 Genetic Analyzer (Applied Biosystems, Foster City, CA, USA).

### 2.6 RT-PCR

The differentially expressed genes identified by sequencing were confirmed by RT-PCR using the specific primer sets (Table 2). The first-strand cDNA was used as a template after normalization with the *β-actin* gene. The PCR amplification protocol with the cDNA of L6 GLUT4myc myocytes was an initial 3 min denaturation at 94 °C, followed by 20–25 cycles of 94 °C for 40 sec, 60 °C for 40 sec, and 72 °C for 40 sec, and a 5 min final extension at 72 °C. The products were purified using a QIAquick gel extraction kit (Qiagen, Alameda, CA, USA) and sequenced (Table 3).

## Figures and Tables

**Table 1:** Identification of differentially expressed genes.

**DEG No.**	**GeneBank Accession No.**	**Identification**	**Expression level in mtDNA depleted cells**
**DEG3**	BC101882.1	**Vesicle amine transport protein 1 homolog**	**increased**
**DEG4**	NM_139098	**DEAD-box helicase 46 (Ddx46)**	**decreased**
**DEG5**	BC083876	**Fermitin family homolog 2**	**increased**
**DEG8**	BQ196300	**cDNA clone UI-R-DQ1-ckv-b-04-0-UI 3′**	**decreased**
**DEG10**	NM_001024268.2	**DNA ligase 1 (Lig1)**	**decreased**
**DEG21**	NM_001013919.1	**Galactokinase 2 (Galk2)**	**increased**
**DEG22**	BC089992.1	**C1q and tumor necrosis factor related protein 5**	**increased**
**DEG23**	AF072892	**Versican V3 isoform precursor**	**decreased**
**DEG25**	BC087063	**Cell division cycle associated 2**	**decreased**
**DEG26**	AI712694	**cDNA clone UI-R-AF1-aau-d-08-0-UI 3′**	**decreased**
**DEG27**	X12367.1	**Glutathione peroxidase**	**decreased**
**DEG28**	NM_199270	**Brain and reproductive organ-expressed (TNFRSF1A modulator) (Bre)**	**increased**
**DEG29**	NM_024396	**ATP binding cassette subfamily A member 2 (Abca2)**	**increased**

**Table 2:** Primers and RT-PCR condition.

**Gene**	**Primer sequence (5′-3′)**		**Product size (bp)**	**Annealing Temperature**	**Concentration**		**Cycle**
					**cDNA**	**Primer**	
**DEG10**	**F.P**	**CCGTGAGAACTTTGTGGAGA**	**580**	**60**	**2** **ng/μl**	**0.5** **μM**	**25**
	**R.P**	**GAACCGAGGGAAACGAAGG**					
**DEG22**	**F.P**	**GTGCCCCCACGATCAGCCTTC**	**301**				
	**R.P**	**AGCGAAGACTGGGGAGCT**					
**DEG26**	**F.P**	**TCTGGAGGACCCACTTTCTG**	**228**				
	**R.P**	**AAACGATGTGATCTACAAAACTGA**					
**β-Actin**	**F.P**	**GCTCCGGCATGTGCAA**	**348**				
	**R.P**	**GCTCATTGTAGAAAGTGTGGTG**					

**Table 3:** Sequences of APCs.

**Gene**	**Primer sequence (5′-3′)**
**ACP1**	**5′-GTCTACCAGGCATTCGCTTCATXXXXXGCCATCGACC-3′**
**ACP2**	**5′-GTCTACCAGGCATTCGCTTCATXXXXXAGGCGATGCC-3′**
**ACP3**	**5′-GTCTACCAGGCATTCGCTTCATXXXXXCCGGAGGATG-3′**
**ACP4**	**5′-GTCTACCAGGCATTCGCTTCATXXXXXGCTGCTCGCG-3′**
**ACP5**	**5′-GTCTACCAGGCATTCGCTTCATXXXXXAGTGCGCTCG-3′**
**ACP6**	**5′-GTCTACCAGGCATTCGCTTCATXXXXXGGCCACATCG-3′**
**ACP7**	**5′-GTCTACCAGGCATTCGCTTCATXXXXXCTGCGGATCG-3′**
**ACP8**	**5′-GTCTACCAGGCATTCGCTTCATXXXXXGGTCACGGAG-3′**
**ACP9**	**5′-GTCTACCAGGCATTCGCTTCATXXXXXGATGCCGCTG-3′**
**ACP10**	**5′-GTCTACCAGGCATTCGCTTCATXXXXXTGGTCGTGCC-3′**
**ACP11**	**5′-GTCTACCAGGCATTCGCTTCATXXXXXCTGCAGGACC-3′**
**ACP12**	**5′-GTCTACCAGGCATTCGCTTCATXXXXXACCGTGGACG-3′**
**ACP13**	**5′-GTCTACCAGGCATTCGCTTCATXXXXXGCTTCACCGC-3′**
**ACP14**	**5′-GTCTACCAGGCATTCGCTTCATXXXXXGCAAGTCGGC-3′**
**ACP15**	**5′-GTCTACCAGGCATTCGCTTCATXXXXXCCACCGTGTG-3′**
**ACP16**	**5′-GTCTACCAGGCATTCGCTTCATXXXXXGTCGACGGTG-3′**
**ACP17**	**5′-GTCTACCAGGCATTCGCTTCATXXXXXCAAGCCCACG-3′**
**ACP18**	**5′-GTCTACCAGGCATTCGCTTCATXXXXXCGGAGCATCC-3′**
**ACP19**	**5′-GTCTACCAGGCATTCGCTTCATXXXXXCTCTGCGAGC-3′**
**ACP20**	**5′-GTCTACCAGGCATTCGCTTCATXXXXXGACGTTGGCG-3′**
